# Analysis of signaling networks distributed over intracellular compartments based on protein-protein interactions

**DOI:** 10.1186/1471-2164-15-S12-S7

**Published:** 2014-12-19

**Authors:** Olga Vasil'evna Popik, Olga Vladimirovna Saik, Evgeny Dmitrievich Petrovskiy, Björn Sommer, Ralf Hofestädt, Inna Nikolaevna Lavrik, Vladimir Aleksandrovich Ivanisenko

**Affiliations:** 1 The institute of Cytology and Genetics, The Siberian Branch of the Russian Academy of Sciences, Novosibirsk, Russia; 2 Bielefeld University, Faculty of Technology, Bioinformatics Department, Bielefeld, Germany; 3 Otto von Guericke University Magdeburg, Medical Faculty, Department Translational Inflammation Research, Magdeburg, Germany; 4 Novosibirsk State University, Novosibirsk, Russia

## Abstract

**Background:**

Biological processes are usually distributed over various intracellular compartments. Proteins from diverse cellular compartments are often involved in similar signaling networks. However, the difference in the reaction rates between similar proteins among different compartments is usually quite high. We suggest that the estimation of frequency of intracompartmental as well as intercompartmental protein-protein interactions is an appropriate approach to predict the efficiency of a pathway.

**Results:**

Using data from the databases STRING, ANDSystem, IntAct and UniProt, a PPI frequency matrix of intra/inter-compartmental interactions efficiencies was constructed. This matrix included 15 human-specific cellular compartments. An approach for estimating pathway efficiency using the matrix of intra/inter-compartmental PPI frequency, based on analysis of reactions efficiencies distribution was suggested. An investigation of KEGG pathway efficiencies was conducted using the developed method. The clusterization and the ranking of KEGG pathways based on their efficiency were performed. "Amino acid metabolism" and "Genetic information processing" revealed the highest efficiencies among other functional classes of KEGG pathways. "Nervous system" and "Signaling molecules interaction" contained the most inefficient pathways. Statistically significant differences were found between efficiencies of KEGG and randomly-generated pathways. Based on these observations, the validity of this approach was discussed.

**Conclusion:**

The estimation of efficiency of signaling networks is a complicated task because of the need for the data on the kinetic reactions. However, the proposed method does not require such data and can be used for preliminary analysis of different protein networks.

## Background

Estimation of efficiency of signaling networks is one of the most relevant problems in the study of biological systems. Analysis of effectiveness of biological networks is needed to meet the challenges of medicine and biotechnology [1,2]. In particular, search for drug targets [3,4], prediction of gene expression [5], engineering of organisms and plant systems [6] can be performed via analysis of various regulatory networks. Common methods for systems analysis of signaling pathways are presented by different modeling approaches, such as flux models [7], kinetic models [8], Boolean models [9,10], Petri net models [11,12] or stochastic modeling methods [13]. Each method has both advantages and limitations. Ordinary Differential Equation (ODE) modeling provides qualitative and quantitative information about processes, though the search of parameters for the reactions is a time-consuming and difficult task. Flux and Boolean models allow steady-state analysis, but do not give a description of the process dynamics. Modeling and analysis using stochastic methods are computationally expensive. All methods require evaluation of reaction parameters, which in turn implies the need for experimental data.

One of the difficulties in modeling a signaling pathway is that biological processes in cells are allocated to different intracellular compartments [14]. Thus, the effectiveness of a pathway can be directly influenced by the distribution of involved proteins over intracellular localizations.

Previously we developed the CELLmicrocosmos PathwayIntegration (CmPI) to support and visualize the subcellular localization prediction of protein-related data such as protein-interaction network [15]. Here, we propose a method for evaluating the pathway efficiency on the basis of data on the intracellular localization of proteins involved in protein-protein interactions (PPI). Current analysis showed that proteins involved in PPI are localized preferably in the same cellular compartment. Moreover, it is shown that Kyoto Encyclopedia of Genes and Genomes (KEGG) pathways [16] significantly differ in efficiency from random pathways. All KEGG pathways have been clustered in eight groups by the distribution of their reactions efficiencies. Clusters statistically differ by average efficiency. Ranking of functional classes of the KEGG pathways based on their efficiency was carried out.

## Results and discussion

### Method for estimating efficiency of signaling pathways

The method for estimating the efficiency of the pathway is based on consideration of PPI frequencies between different intracellular compartments. We assume that if PPI in general occurs more frequently between proteins from particular compartments, the interactions which contain proteins located in these compartments would be more effective within the pathways. Thus, the optimality of a pathway reaction distribution over the intracellular localization may reflect the efficiency of the pathway, with the most optimal distribution being the one where the frequency of observed interactions between proteins localized in intracellular compartments involved in the pathway has a maximum value.

To analyze the effectiveness of intra/inter-compartmental interactions, 15 major locations of eukaryotic cells were selected: Cytoplasm, Nucleus, Secreted, Membrane, Chromosome, Endoplasmic reticulum, Golgi apparatus, Endosome, Lysosome, Mitochondrion, Cell junction, Lipid-anchor, Cell projection, Peroxisome and cytoplasmic vesicle. The localizations were selected by following rules. We considered only the highest hierarchy level of localizations presented in UniProt [17], data on underlying in hierarchy localizations were added to localizations with the highest hierarchy level. Finally we took 15 localizations containing more than 200 numbers of proteins with available PPI data. We used data on 16,000 human proteins with the information about their compartmentalization (Figure S1). For this group of proteins, 600,000 cases of PPI were reported.

On the basis of these data we find efficiency of a reaction and a molecular-genetic network by following approach:

Let $L_{i}$, $L_{j}$ be compartments *i *and *j*, $m_{i}$ and $m_{j}$ be the numbers of proteins that are localized in compartments $L_{i}$ and $L_{j}$, correspondingly. $P_{L_{i},L_{j}}$ - the number of interactions between all proteins from $L_{i}$ and all proteins from $L_{j}$found in the databases. Then, the efficiency of any molecular reaction of proteins localized in $L_{i}$ and $L_{j}$ is calculated as follows:

$$E_{L_{i},L_{j}}=\frac{P_{L_{i},L_{j}}}{m_{i}*m_{j}},$$

$E_{L_{i},L_{j}}$ is a symmetric matrix (Additional file 1 Table S1). The efficiencies $E_{L_{k},L_{k}}$ of reactions occurring in the same compartment $L_{k}$ are presented on the diagonal of the matrix. The efficiencies $E_{L_{i},L_{j}}$ reflect efficiencies of reactions of proteins from different localizations $L_{i}$ and $L_{j}$, i≠j. In most cases diagonal elements $E_{L_{k},L_{k}}$ have higher values in comparison with other elements from the row $E_{L_{k},L_{j}}\left(j\neq k\right)$ or column $E_{L_{i},L_{k}}\left(i\neq k\right)$. It can be observed that reactions of proteins from the one compartment take place in more efficient way than reactions of proteins from different compartments. The only exception is the membrane compartment. In this case the diagonal element is the smallest compared to other compartments.

The efficiency EQ of a molecular-genetic network Q involving N reactions is defined as a function of efficiencies of the reactions: $\text{EQ}=\text{E}{\text{Q}}_{q\in P}\left(E_{L\left[R_{q}\left[P_{1}\right]\right],L\left[R_{q}\left[P_{2}\right]\right]}\right)$, where in case of PPI, $R_{q}$ is the reaction number *q *of the network Q, $P_{1}$ and $P_{2}$ are proteins, reacting in $R_{q}$. In case of not PPI, we consider proteins $P_{1}$ and $P_{2}$ from reactions $R_{q}$ and $R_{q+1}$.

$L\left[R_{q}\left[P_{1}\right]\right]\text{and}L\left[R_{q}\left[P_{2}\right]\right]$ - are localizations of proteins $P_{1}$ and $P_{2}$, correspondingly. Thus we can estimate the statistical significance of difference between analyzed networks efficiencies and random networks based on distribution of reaction efficiencies. To compare molecular-genetic networks between each other we can use either distribution or mean value of reaction efficiencies:

$$\text{Eff}=\frac{\sum_{\text{q}=1}^{N}k_{L\left[R_{q}\left[P_{1}\right]\right],L\left[R_{q}\left[P_{2}\right]\right]}}{N}$$

### KEGG pathways analysis

There were 282 KEGG human pathways analyzed, including totally 50.000 reactions (Additional file 2 Table S2).

**Figure 1:**
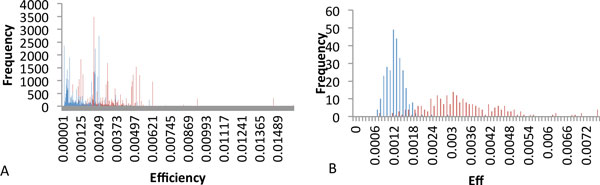
**Comparison of KEGG and random pathways by distribution of reaction efficiencies (A) and the mean efficiency of pathways (B)**. Random pathways are shown in blue. KEGG pathways are shown in red.

On the first step, efficiency distributions of all reactions from KEGG pathways were compared with the same distribution for "random reactions" (Figure 1A). Random reactions are obtained by permutation of KEGG reactions, in which we randomly replaced proteins by ones from list of all proteins from KEGG pathways. These two efficiency distributions have statistically significant difference by the chi square test [p-value <10E-16]. The average efficiency of the reactions from KEGG pathways exceeds two times the one of random reactions.

To compare the mean efficiency (Eff) of KEGG pathways with the one of random pathways, Eff distributions of 282 KEGG pathways and more than 10000 random pathways were calculated. Random pathways were generated by permutation of KEGG pathways in following way: for each KEGG pathway we generated 1000 of random pathways by replacing the proteins in each reaction by randomly chosen ones from the list of all KEGG proteins. If one protein is involved in several reactions of the pathway - we replace it in all these reactions by the same random protein. It was found that the Eff distribution of KEGG pathways (Figure 1B) has a statistically significant difference over the Eff distribution of random pathways using chi square test (p-value <10E-5).

Also, it was important to check whether there is a correlation between the length of the pathways and their efficiency. The value of Pearson correlation coefficient was equal to R =- 0.1 (p-value <0.01). The value of R was low, so we cannot make any concrete conclusions. However, it is negative, suggesting a weak reciprocal relationship with the length of the pathways.

**Figure 2:**
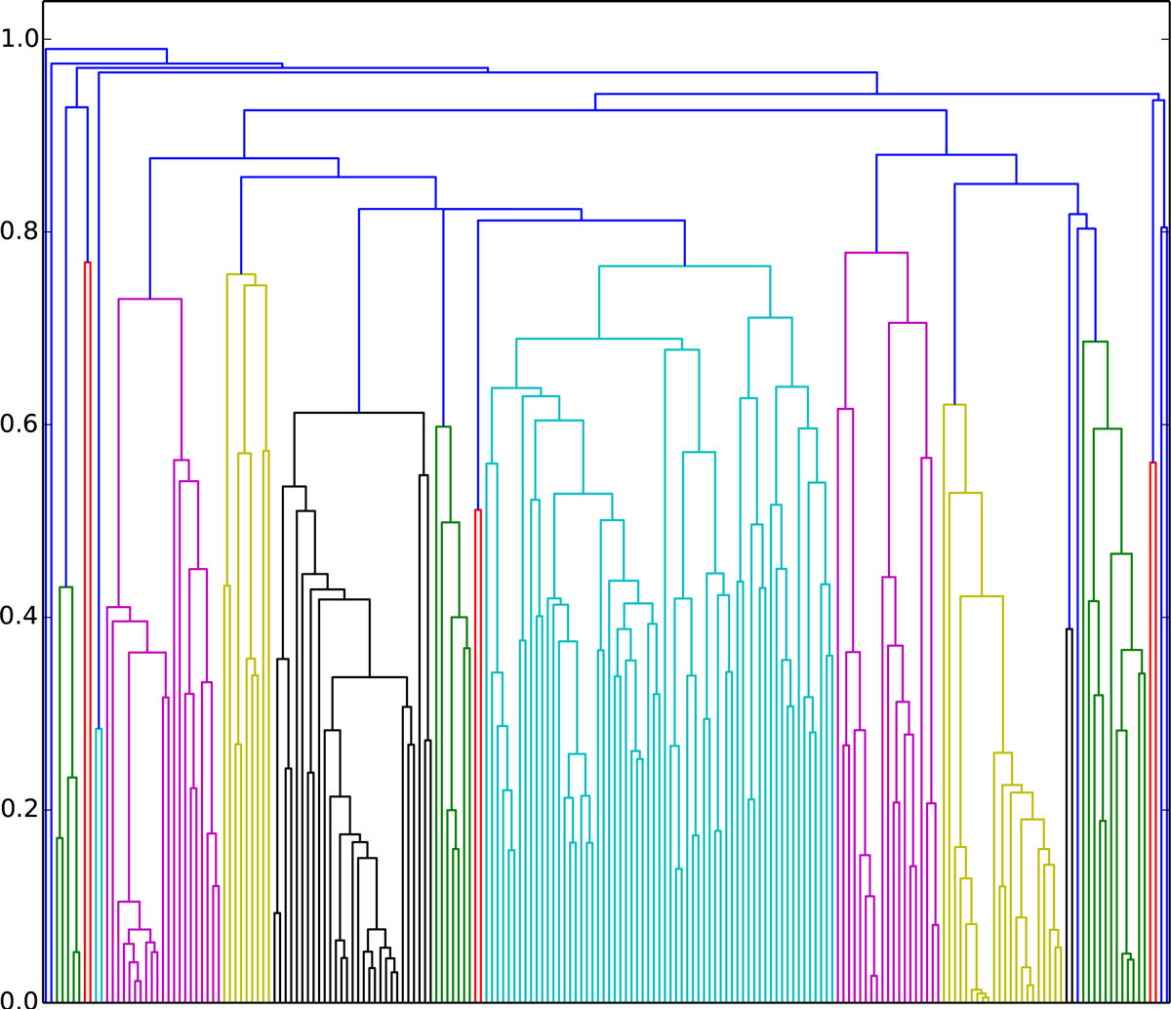
**The dendogram of all KEGG pathways, clustered by the correlation distance between distributions of pathways reactions efficiencies**.

To identify similar KEGG pathways, hierarchical clustering was performed on the basis of the correlation distance between pairs of pathways (Figure 2). The correlation distance between a single pair of KEGG pathways was calculated as Pearson correlation between a pair of distributions of pathways reactions efficiencies.

**Figure 3:**
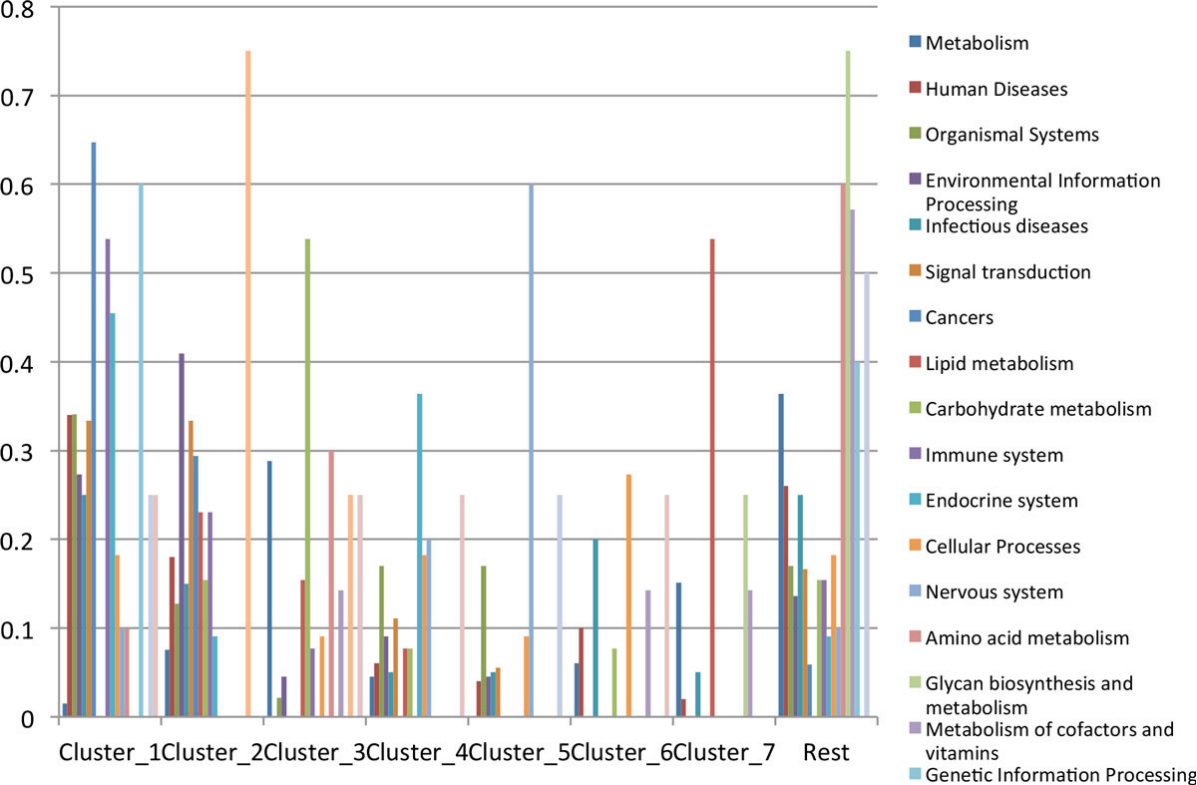
**Distribution of KEGG functional classes over clusters (ratio)**.

Seven major clusters can be identified in the dendogram presented in Figure 2. These clusters significantly differ by the distribution of the mean efficiencies (Eff) of the pathways (Additional file 1 Figure S2). Distribution of functional classes of KEGG pathways over clusters is shown in Figure 3. There are several classes that mainly lay in one of the clusters. For example, "Cancers", "Immune systems", "Genetic Information Processing" and "Endocrine system" are represented in the cluster 1 (more than 50% of all the pathways of each class). Pathways that are included in these classes have the similar efficiency. Another group is represented by pathways of the "Signaling molecules and interaction" and "Environmental Information Processing" classes, which appear in the cluster 2. Clusters 3, 5 and 7 are mainly presented by unique classes, thus "Carbohydrate metabolism" belongs to the cluster 3, "Nervous system" could be assigned to the cluster 5 and "Amino acid metabolism" could be assigned to the cluster 7. "Metabolism" could be classified as consisting of pathways with the most diverse efficiencies, thus this class is about equally represented in all clusters.

KEGG functional classes of pathways were ranked by the mean efficiency of included pathways. The highest efficiency is observed within pathways from "Amino acid metabolism", "Genetic information processing" and "Carbohydrate metabolism" classes.

**Figure 4:**
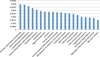
**Mean efficiencies of pathways from functional classes**.

"Metabolism" class consists of various subclasses, therefore it is located in the middle of the list. The less effective pathways include "Lipid metabolism", "Nervous system" and "Signaling molecules interaction" classes. Despite the fact that the average efficiency varies slightly between some classes (Figure 4), the distributions of efficiencies of reactions involved in pathways from the functional classes differ more (Additional file 1 Figure S3), as localizations of reactions are taken into account. This can explain the allocation of different functional classes of the KEGG pathways in different clusters.

## Conclusion

Evaluation of the efficiency of the signaling networks currently remains an important issue. The method for preliminary analysis of networks lacking the data on kinetic parameters was suggested to avoid one of the main obstacles on the way to practical application of existing methods for modeling the dynamic of the molecular genetic systems. The matrix of intra/inter-compartmental interactions efficiencies was constructed for 15 specific human cellular localizations based on PPI data and data on protein distribution over cellular compartments. The analysis of the matrix revealed that the frequency of PPI of proteins from the same compartment is higher in comparison to frequency of PPI of proteins from different compartments. A new method for evaluating pathway efficiency was proposed; all KEGG human pathways were estimated by mean efficiency and clustered based on correlation distances between the distributions of pathway reaction efficiencies. The distribution of pathway functional classes over clusters shows that some classes are mainly presented in one cluster.

The proposed method can be used for the preliminary analysis of the effectiveness of various signaling networks, including networks, for which there is not enough data for modeling them with more accurate methods.

## Material and methods

PPI data was extracted from STRING [18], IntAct [19], and ANDSystem [20]. STRING is a database containing known and predicted protein interactions. The interactions include direct (physical) and indirect (functional) associations. IntAct is a database containing protein-protein interaction data. All interactions are derived from literature curation or direct user submissions. The ANDSystem is designed to reconstruct and analyze associative gene networks. The ANDSystem incorporates utilities for automated knowledge extraction from Pubmed-published scientific texts, and analysis of information from various databases. In addition, the ANDCell database contains information on molecular-genetic events retrieved from texts and databases. Data on subcellular localization of human proteins was extracted from ANDSystem that contains - in addition to the text mining-based information - also data from the UniProt database. The classification of the pathways by their efficiency was conducted on a set of pathways from the KEGG database. 282 human pathways were analyzed (Release 70.1, June 1, 2014).

## Competing interests

The authors declare that they have no competing interests.

## Authors' contributions

OVP, OVS and VAI conceived the method. INL and RH carried out expert assessment and interpretation of results. OVP and OVS implemented the method and BS tested the method. EDP performed the statistical analyses. OVP and VAI drafted the manuscript. VAI supervised the whole studies. All authors read, corrected and approved the final manuscript

## Supplementary Material

Additional file 1 Click here for file

Additional file 2 Click here for file

## Declarations

Publication of this article has been funded by Russian Science Foundation grant No 14-44-00011.

This article has been published as part of *BMC Genomics *Volume 15 Supplement 12, 2014: Selected articles from the IX International Conference on the Bioinformatics of Genome Regulation and Structure\Systems Biology (BGRS\SB-2014): Genomics. The full contents of the supplement are available online at http://www.biomedcentral.com/bmcgenomics/supplements/15/S12.
