# Supplementary material for: Analysis of signaling networks distributed over intracellular compartments based on protein-protein interactions
Source: BMC Genomics. 2014 Dec 19;15(Suppl 12):S7. doi: 10.1186/1471-2164-15-S12-S7 (PMC4303950; doi:10.1186/1471-2164-15-S12-S7)
Supplement: Additional file 1 [file 1471-2164-15-S12-S7-S1.docx]

Supplementary Text

**Efficiency analysis of molecular-genetic networks with function distributed over intracellular components**

O. Popik, O. Saik, E. Petrovskiy, B. Sommer, R. Hofestädt, V. A. Ivanisenko

The matrix of intercompartmental interaction efficiencies for 15 human cellular components: Cytoplasm, Nucleus, Secreted, Membrane, Chromosome, Endoplasmic reticulum, Golgi apparatus, Endosome, Lysosome, Mitochondrion, Cell junction, Lipid-anchor, Cell projection, Peroxisome, Cytoplasmic vesicle.

|  | Cytoplasm | Nucleus | Secreted | Membrane | Chromosome | Endoplasmic reticulum | Golgi apparatus | Endosome | Lysosome | Mitochondrion | Cell junction | Lipid-anchor | Cell projection | Peroxisome | Cytoplasmic vesicle |
| --- | --- | --- | --- | --- | --- | --- | --- | --- | --- | --- | --- | --- | --- | --- | --- |
| Cytoplasm | 0.00515 | 0.00447 | 0.00421 | 0.00147 | 0.00412 | 0.00316 | 0.00360 | 0.00411 | 0.00397 | 0.00318 | 0.00386 | 0.00367 | 0.00411 | 0.00293 | 0.00372 |
| Nucleus | 0.00447 | 0.00537 | 0.00389 | 0.00118 | 0.00562 | 0.00273 | 0.00294 | 0.00330 | 0.00301 | 0.00270 | 0.00293 | 0.00277 | 0.00300 | 0.00241 | 0.00295 |
| Secreted | 0.00421 | 0.00389 | 0.01466 | 0.00222 | 0.00241 | 0.00392 | 0.00414 | 0.00442 | 0.00783 | 0.00331 | 0.00411 | 0.00478 | 0.00429 | 0.00353 | 0.00522 |
| Membrane | 0.00147 | 0.00118 | 0.00222 | 0.00076 | 0.00085 | 0.00149 | 0.00165 | 0.00196 | 0.00234 | 0.00123 | 0.00153 | 0.00185 | 0.00150 | 0.00119 | 0.00137 |
| Chromosome | 0.00412 | 0.00562 | 0.00241 | 0.00085 | 0.02089 | 0.00201 | 0.00209 | 0.00253 | 0.00238 | 0.00217 | 0.00236 | 0.00195 | 0.00269 | 0.00108 | 0.00188 |
| Endoplasmic reticulum | 0.00316 | 0.00273 | 0.00392 | 0.00149 | 0.00201 | 0.00504 | 0.00411 | 0.00391 | 0.00528 | 0.00266 | 0.00259 | 0.00268 | 0.00281 | 0.00314 | 0.00299 |
| Golgi apparatus | 0.00360 | 0.00294 | 0.00414 | 0.00165 | 0.00209 | 0.00411 | 0.00566 | 0.00527 | 0.00576 | 0.00251 | 0.00334 | 0.00353 | 0.00367 | 0.00252 | 0.00402 |
| Endosome | 0.00411 | 0.00330 | 0.00442 | 0.00196 | 0.00253 | 0.00391 | 0.00527 | 0.00973 | 0.00920 | 0.00243 | 0.00403 | 0.00462 | 0.00433 | 0.00240 | 0.00477 |
| Lysosome | 0.00397 | 0.00301 | 0.00783 | 0.00234 | 0.00238 | 0.00528 | 0.00576 | 0.00920 | 0.02330 | 0.00352 | 0.00307 | 0.00483 | 0.00377 | 0.00350 | 0.00412 |
| Mitochondrion | 0.00318 | 0.00270 | 0.00331 | 0.00123 | 0.00217 | 0.00266 | 0.00251 | 0.00243 | 0.00352 | 0.00630 | 0.00220 | 0.00236 | 0.00227 | 0.00315 | 0.00234 |
| Cell junction | 0.00386 | 0.00293 | 0.00411 | 0.00153 | 0.00236 | 0.00259 | 0.00334 | 0.00403 | 0.00307 | 0.00220 | 0.00885 | 0.00327 | 0.00481 | 0.00166 | 0.00340 |
| Lipid-anchor | 0.00367 | 0.00277 | 0.00478 | 0.00185 | 0.00195 | 0.00268 | 0.00353 | 0.00462 | 0.00483 | 0.00236 | 0.00327 | 0.01437 | 0.00436 | 0.00322 | 0.00352 |
| Cell projection | 0.00411 | 0.00300 | 0.00429 | 0.00150 | 0.00269 | 0.00281 | 0.00367 | 0.00433 | 0.00377 | 0.00227 | 0.00481 | 0.00436 | 0.00561 | 0.00194 | 0.00329 |
| Peroxisome | 0.00293 | 0.00241 | 0.00353 | 0.00119 | 0.00108 | 0.00314 | 0.00252 | 0.00240 | 0.00350 | 0.00315 | 0.00166 | 0.00322 | 0.00194 | 0.01786 | 0.00223 |
| Cytoplasmic vesicle | 0.00372 | 0.00295 | 0.00522 | 0.00137 | 0.00188 | 0.00299 | 0.00402 | 0.00477 | 0.00412 | 0.00234 | 0.00340 | 0.00352 | 0.00329 | 0.00223 | 0.00708 |

Table S1 The matrix of intercompartmental interaction efficiencies. Higher values are colored more red.

The distribution of all proteins over compartments was constructed on data from ANDSystem. Despite the fact that the largest number of proteins is found in the membrane – the coefficient of efficiency of PPI between membrane proteins is very low (Table S1), lower than the coefficients of the efficiency of interaction between membrane proteins and proteins from other components

Figure S1 Distribution of proteins over compartments.

All KEGG human pathways were downloaded from KEGG database and the efficiencies of the pathways were computed using the suggested approach (Table S2).

Hierarchical clustering of KEGG pathways was performed on the basis of the correlation distance between pathway pairs. The correlation distance between the pair of KEGG pathways was calculated as Pearson correlation between a pair of distributions of pathways reaction efficiencies. Distributions of mean efficiencies of pathways clustered in the same cluster were built. Pair-wise distribution comparison by chi square test of the distributions revealed that there is statistically significant difference between clusters.


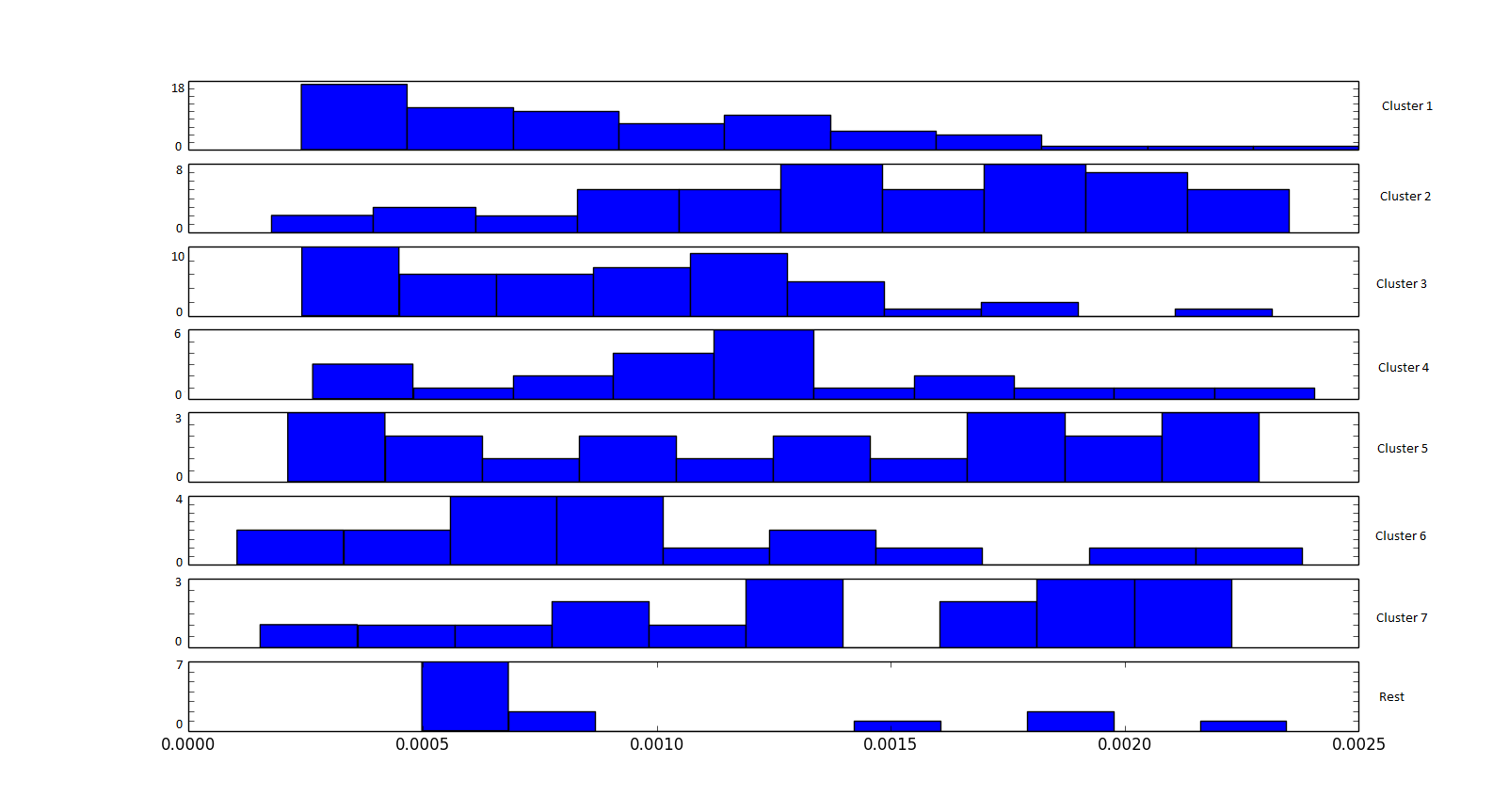


Figure S2 Distribution of mean efficiencies of pathways over clusters.

The 20 largest functional pathway classes from all KEGG functional classes were studied: amino acid metabolism, genetic information processing, carbohydrate metabolism, infectious diseases, cellular processes, metabolism, cell communication, endocrine system, metabolism of cofactors and vitamins, human diseases, cancers, signal transduction, immune system, environmental information processing, substance dependence, glycan biosynthesis and metabolism, organismal systems, lipid metabolism, signaling molecules and interaction, nervous system (Figure S3).


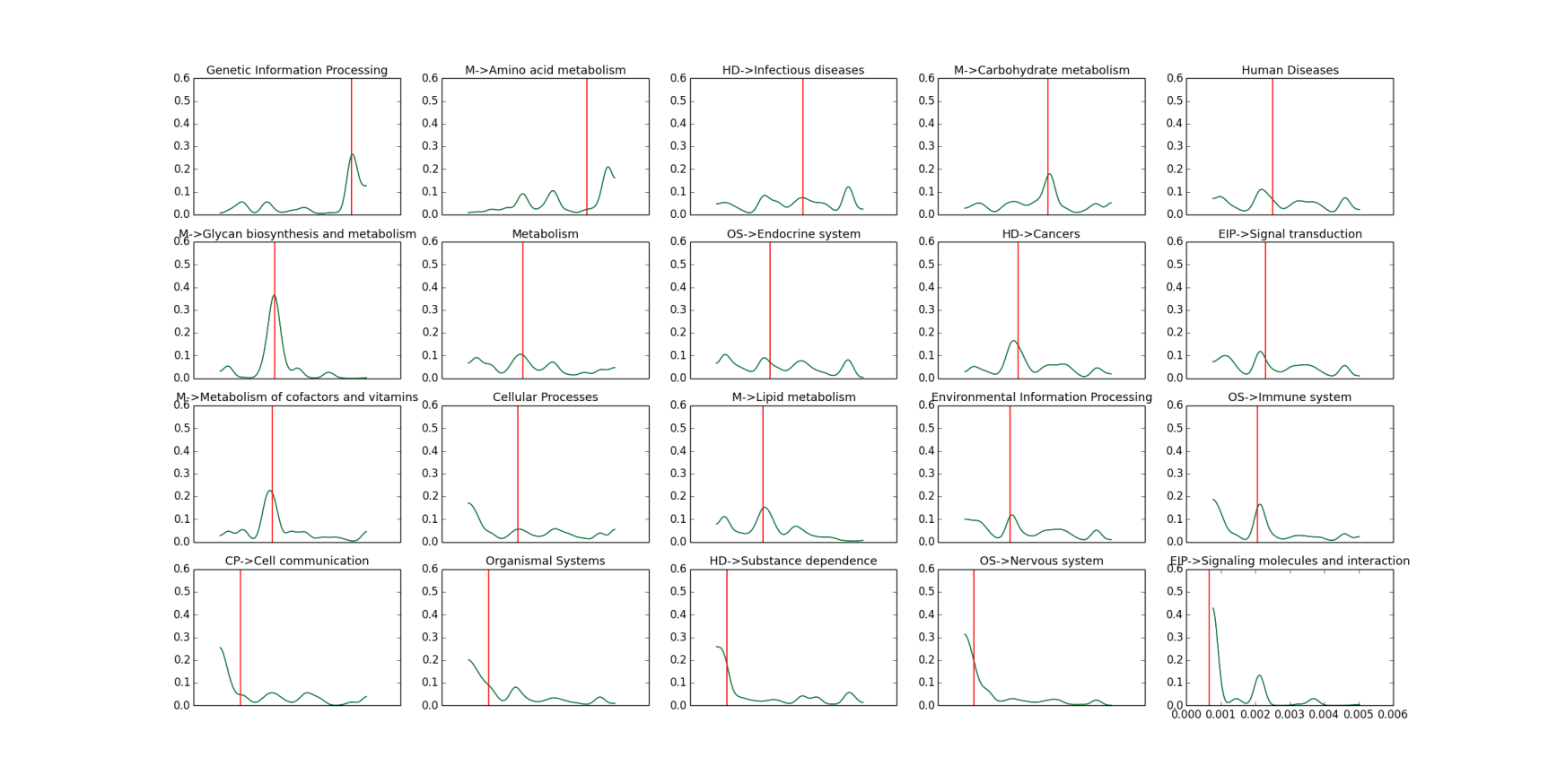


Figure S3 The 20 largest functional pathway classes from all KEGG functional classes sorted by median efficiency value.
